# Treatment With an Osteochondral Graft From the Olecranon for a Fernandez Classification Type 4 Distal Radial Fracture

**DOI:** 10.1016/j.jhsg.2025.01.015

**Published:** 2025-02-24

**Authors:** Yusuke Yuasa, Seietsu Senma, Koji Nozaka, Tsuyoshi Shirahata, Hikaru Saito, Naohisa Miyakoshi

**Affiliations:** ∗ Department of Orthopedic Surgery, Akita University Graduate School of Medicine, Akita, Japan; † Department of Orthopedic Surgery, Nakadori General Hospital, Akita, Japan

**Keywords:** Articular defect, Distal Radial Fracture, Olecranon graft, Osteochondral grafting, Radiocarpal dislocation

## Abstract

Fernandez type 4 distal radial fractures involve radiocarpal dislocation with an avulsion fracture of the articular margin, typically causing considerable instability because of ligament and joint capsule injuries. We present a 30-year-old man with a left distal radial fracture and dorsal radiocarpal dislocation because of a motor vehicle accident. Initial reduction, internal fixation, and ligament repair failed to prevent recurrent dislocation. A second surgery was performed to address a dorsal articular surface defect in the lunate fossa using an osteochondral graft from the olecranon tip, stabilized with Kirschner wires and an external fixator. Fixation was removed after 4 weeks, followed by range of motion exercises. At 3 months, graft union with good articular congruency was confirmed, with improved wrist function by 15 months. This case highlights the efficacy of olecranon osteochondral grafting for articular defects in unstable distal radial fractures, emphasizing the importance of early intervention and appropriate fixation for optimal outcomes.

The Fernandez classification categorizes distal radial fractures based on injury mechanisms.^1^ Type 4 fractures are characterized by radiocarpal dislocation with an avulsion fracture of the radiocarpal articular margin. In addition to the wrist fracture-dislocation, these fractures involve injuries to the radiocarpal ligament and joint capsule, making them highly unstable. In most cases, stabilization can be achieved through bony fixation and ligament repair. However, we report a case of a Fernandez type 4 distal radial fracture that required an olecranon osteochondral graft to restore joint stability. The patient was informed of the intention to publish data from this case and provided his consent.

## Case Report

**Figure 1:**
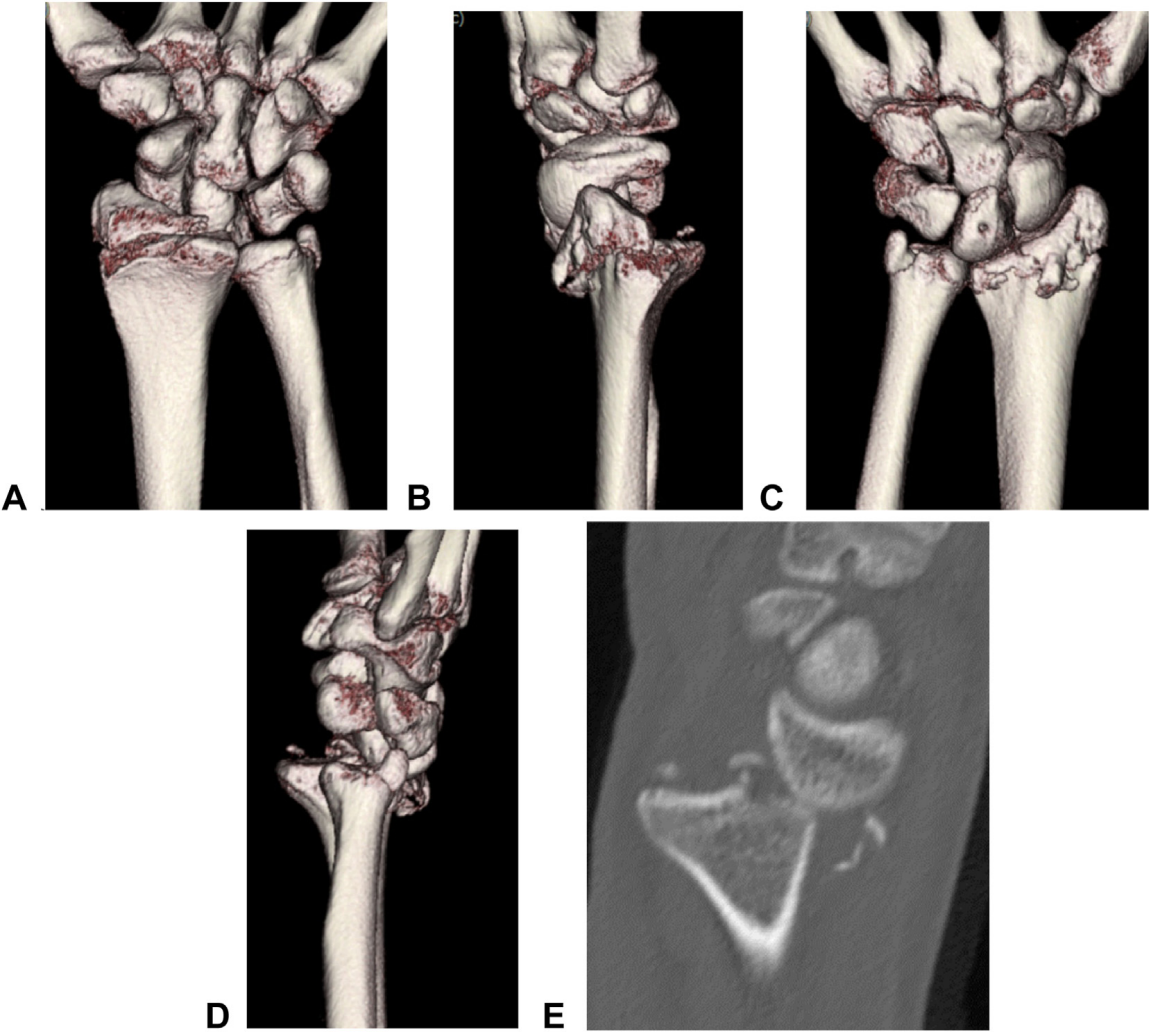
Three-dimensional computed tomographic reconstructions of the left wrist. **A** Palmar view. **B** Radial view. **C** Dorsal view. **D** Ulnar view. **E** Sagittal reformation of computed tomography confirms a distal radial fracture, including a radial styloid fracture, dorsal rim avulsion fracture, and dorsal dislocation of the radiocarpal joint.

**Figure 2:**
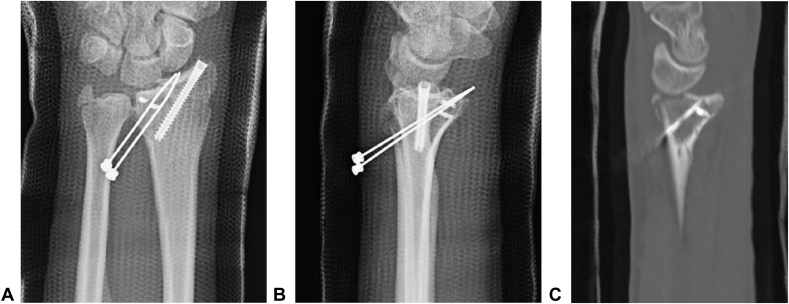
Recurrence of the dorsal radiocarpal joint dislocation. **A** Anteroposterior radiograph. **B** Lateral radiographs. **C** Computed tomography sagittal reformation.

A 30-year-old man sustained multiple injuries in a motor vehicle collision with a truck and was transported to a general hospital. The injuries included sternal and rib fractures, bilateral pulmonary contusions, a right internal thoracic artery injury, and a right subtrochanteric femoral fracture. The patient also experienced a left distal radial fracture and presented in shock. On the day of injury, transcatheter arterial embolization was performed to control the internal bleeding from the thoracic artery, achieving hemostasis and stable vital signs. Three days postinjury, open reduction and internal fixation of the right subtrochanteric femoral fracture were performed. Computed tomography (CT) of the left wrist revealed a radial styloid fracture, dorsal rim avulsion fracture, and dorsal radiocarpal joint dislocation (Fig. 1), consistent with Fernandez type 4 injury. Surgery was performed 2 weeks after the injury, involving closed reduction of the dorsal dislocation, followed by open reduction and internal fixation of the radial styloid fracture, as well as repair of the palmar radiocarpal ligament through a palmar approach. However, dorsal dislocation of the radiocarpal joint gradually recurred (Fig. 2), prompting referral to our hospital for further treatment.

Three weeks after surgery, an open reduction of the dorsal dislocation was performed through a dorsal approach. Intraoperatively, no suitable bony fragments for fixation were found, and a dorsal bone defect of the lunate fossa articular surface was observed. Kirschner wires were inserted for joint fixation, and an external fixator was applied to maintain the reduction. A postoperative CT scan confirmed adequate reduction and provided a clearer view of the dorsal bone defect on the lunate fossa.

**Figure 3:**
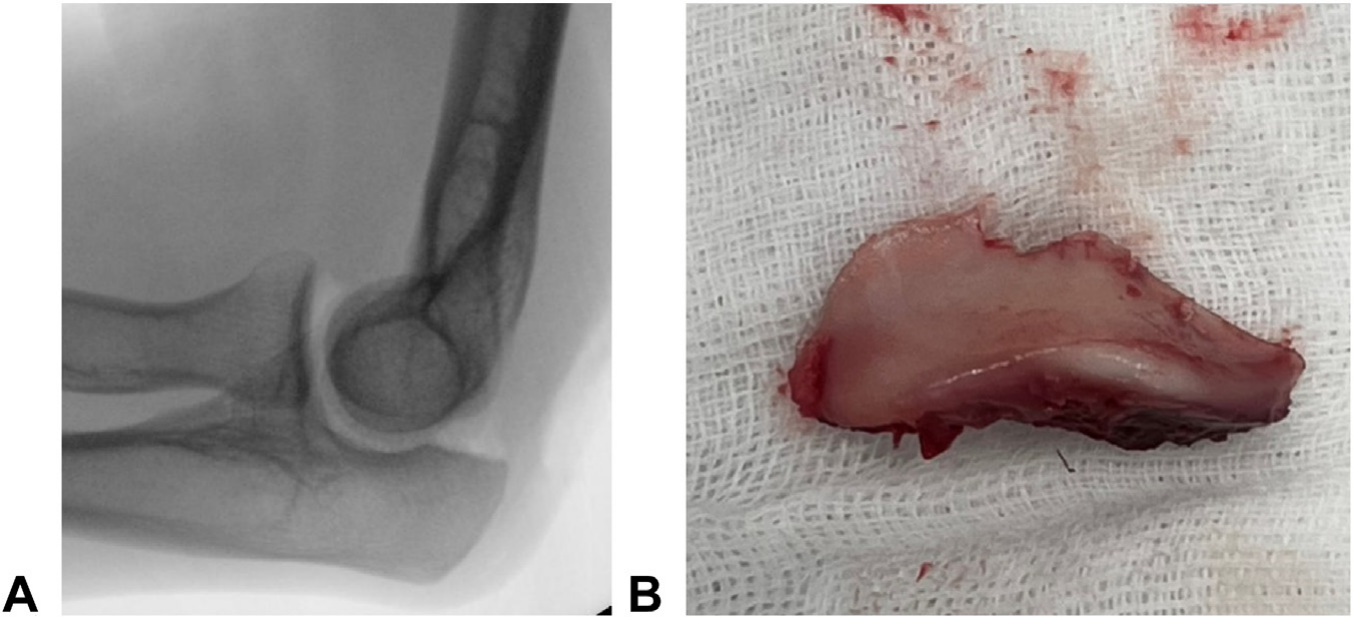
Harvesting of the osteochondral graft from the tip of the olecranon.

**Figure 4:**
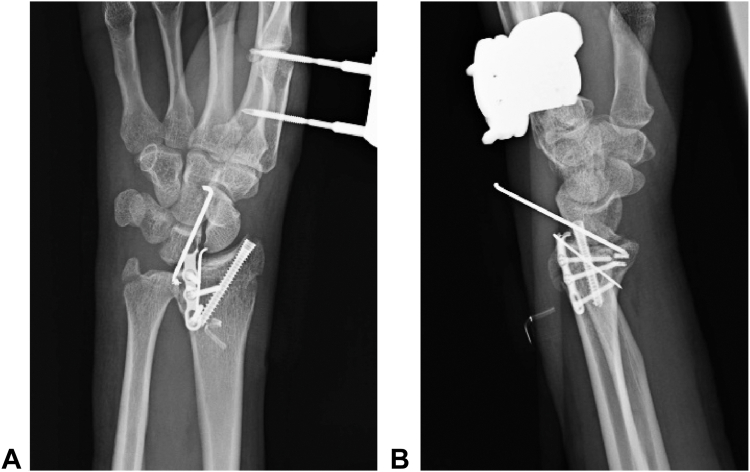
The graft is fixed with a plate and Kirschner wire. **A** Post-transplant anteroposterior radiograph. **B** Post-transplant lateral radiograph.

**Figure 5:**
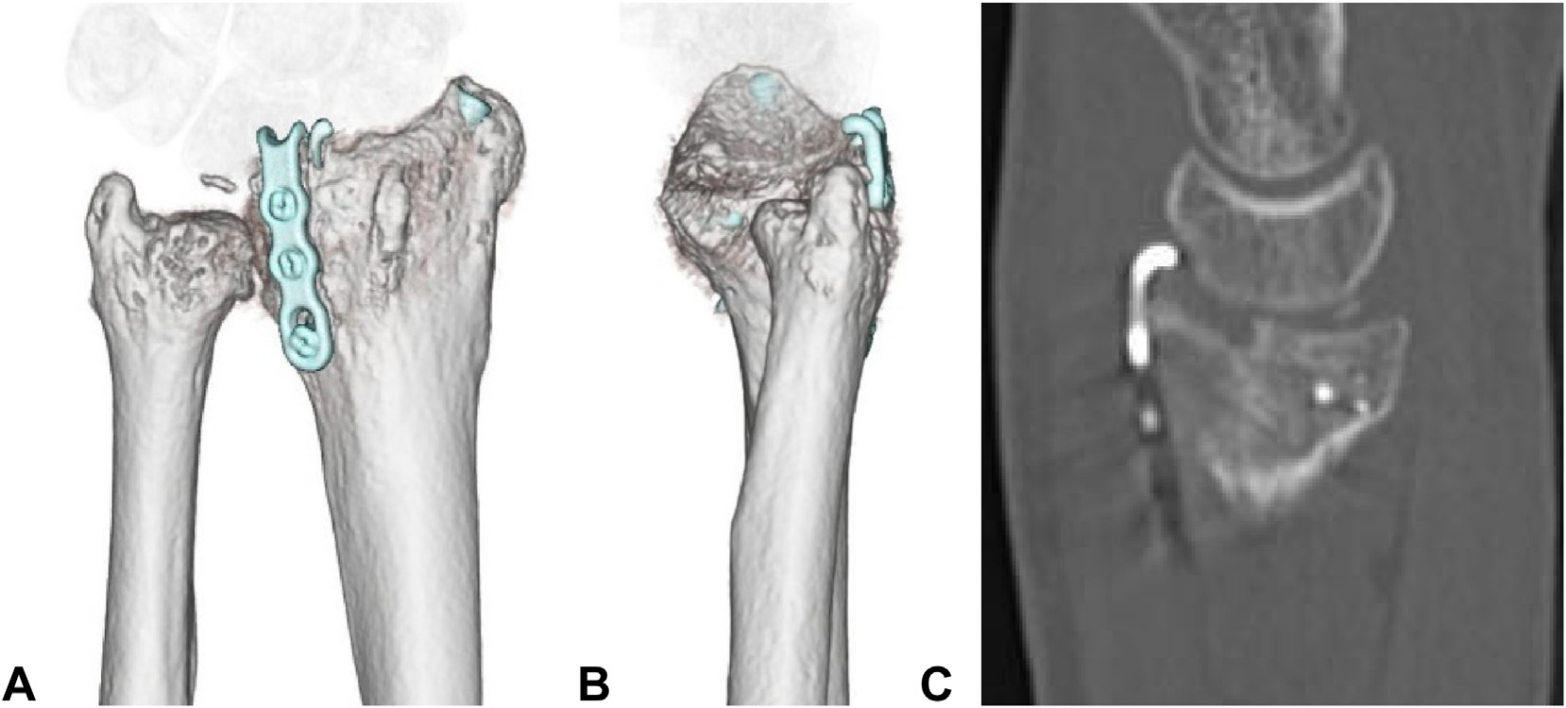
Images showing graft union and good articular surface congruency. **A** Post-transplant dorsal view. **B** Post-transplant ulnar view. **C** Computed tomography sagittal reformation.

**Figure 6:**
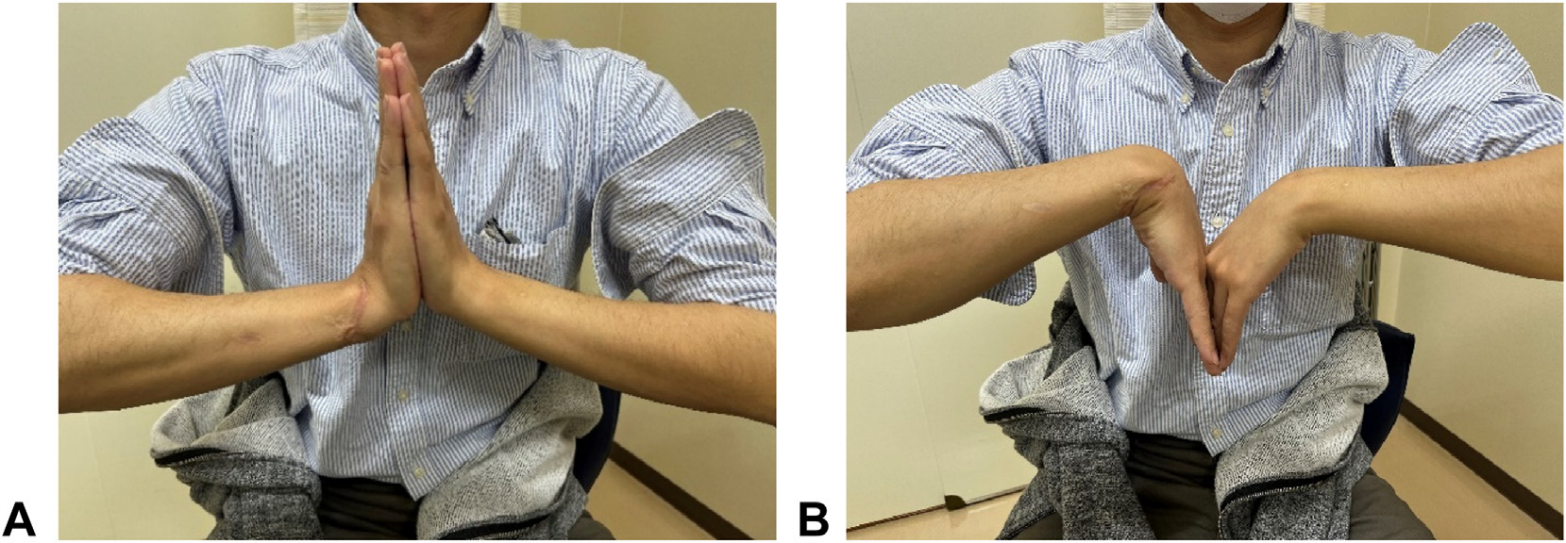
Wrist function 1 year and 3 months after olecranon osteochondral graft surgery. **A** Extension. **B** Flexion.

Given the recurrent dislocation caused by the bone defect, bone support was deemed necessary. Considering the patient’s young age, cartilage reconstruction of the articular surface was considered the optimal option. An osteochondral graft was harvested from the tip of the olecranon (Fig. 3) and shaped to fit the defect. The stability of the elbow at the donor site was confirmed via fluoroscopy. The graft was fixed in place with a plate and a Kirschner wire, and additional Kirschner wires and an external fixator were used to protect the graft (Fig. 4). At 4 weeks after surgery, the external fixator and Kirschner wires were removed, followed by initiation of range of motion exercises. A CT scan at 3 months post-grafting revealed graft union with good articular congruency (Fig. 5). At the final follow-up 1 year and 3 months postsurgery, the patient demonstrated considerable recovery in wrist motion, achieving 45° flexion, 55° extension, 95° supination, and 55° pronation without reported pain (Fig. 6). He returned to work with a Hand20 score of 7 and a *Quick*DASH (Disabilities of the Arm, Shoulder, and Hand) score of 6.8 for disability/symptoms and 0 for work-related limitations.

## Discussion

Radiocarpal fracture dislocations are exceedingly rare, accounting for only 0.2% of all dislocations.^2^ These injuries typically result from high-energy trauma, such as motor vehicle accidents.^3^^,^^4^ In this case, the patient sustained the injury in a motor vehicle accident.

Type 4 of the Fernandez classification involves radiocarpal dislocation and is associated with damage to the radiocarpal ligaments and joint capsule. Therefore, this type of distal radial fracture is characterized by considerable instability. Yuan et al^4^ documented that posttraumatic arthrosis and progressive ulnar subluxation of the carpus occurred in 31% and 23% of wrists, respectively, according to the most recent radiographs. For this type of dislocation fracture, reduction of the dislocation and fixation of the fracture is recommended, followed by repair of the palmar radiocarpal ligaments if the instability persists. Moreover, previous study have suggested that temporary joint fixation using Kirschner-wires may be necessary after ligament repair to maintain stability.^5^ In the present case, temporary joint fixation was not performed after the palmar radiocarpal ligament was repaired. Although such fixation might be necessary to protect the ligament, the large bone defect in the articular surface likely would have resulted in persistent instability, even with adequate protection of the ligament.

Previous reports have described the reconstruction of the articular surface of the distal radius using a pedicled pisiform graft and a free vascularized fibular head graft.^6^^,^^7^ The pedicled pisiform graft offers several advantages, including a low donor-site morbidity rate, no requirement for microsurgical vascular anastomosis, as well as a relatively large and anatomically constant pedicle that is sufficiently long to reach the distal radius. However, a limitation of this technique is the restricted size of the cartilage defect that can be reconstructed with the pisiform, which is approximately 15 × 10 mm.^6^ The vascularized fibular head graft is suitable for the reconstruction of relatively large articular surfaces. However, disadvantages of this technique include the complexity of the procedure because of the need for microsurgical vascular anastomosis and the risk of complications, such as peroneal nerve palsy.^7^ Although both techniques allow for the reconstruction of the articular surface with cartilage, a common disadvantage is their inability to restore the original concave shape of the articular surface.

In this case, a bony defect was present on the dorsal articular surface of the lunate fossa. Considering the patient’s young age, cartilage reconstruction was deemed ideal to restore the original concave articular surface, and an osteochondral graft from the olecranon tip was selected as a suitable option, given the limitations of other graft choices. Previous studies, including those by Moritomo et al^8^ and Bianco et al,^9^ have shown favorable outcomes with olecranon tip grafts in coronoid process reconstruction. Bell et al^10^ reported that resection of 50% of the articular surface of the olecranon increased the varus instability of the elbow joint, whereas resection of 87.5% increased the valgus instability. In this case, approximately 25% of the articular surface of the olecranon was resected. Although no considerable instability was observed, minor elbow instability remains a possibility warranting long-term follow-up.

Dumontier et al^5^ reported a 35% reduction in wrist range of motion following this type of injury. In this case, a 33.3% reduction in wrist range of motion was observed, showing similar limitations. We recommend early surgical intervention and prompt initiation of the range of motion exercises to improve outcomes. Although a previous study reported a *Quick*DASH (Disabilities of the Arm, Shoulder, and Hand) score of 29.5, the patient in this case demonstrated a more favorable score of 6.8, suggesting a satisfactory clinical course.^3^

A limitation of this case is the short follow-up period of approximately 1 year. Additionally, the possibility of bone resorption in the future cannot be ruled out, as the osteochondral graft from the olecranon tip is not vascularized.

We performed osteochondral grafting from the olecranon tip to address an articular surface defect of the distal radius caused by a Fernandez type 4 distal radial fracture. The patient had a favorable outcome with improvements in function and range of motion, suggesting that this surgical technique may offer a viable treatment option in similar cases.

## Conflicts of Interest

No benefits in any form have been received or will be received related directly to this article.
